# Impact of male circumcision on risk of HIV infection in men in a changing epidemic context – systematic review and meta‐analysis

**DOI:** 10.1002/jia2.25490

**Published:** 2020-06-18

**Authors:** Timothy MM Farley, Julia Samuelson, M Kate Grabowski, Wole Ameyan, Ronald H Gray, Rachel Baggaley

**Affiliations:** ^1^ Sigma3 Services SÀRL Nyon Switzerland; ^2^ Global HIV, Hepatitis and STIs Programmes World Health Organization Geneva Switzerland; ^3^ Department of Pathology Johns Hopkins School of Medicine Baltimore MD USA; ^4^ Department of Epidemiology Johns Hopkins Bloomberg School of Public Health Baltimore MD USA; ^5^ Rakai Health Sciences Program Kalisizo Uganda

**Keywords:** circumcision male, HIV incidence, HIV prevention, meta‐analysis, review

## Abstract

**Introduction:**

WHO/UNAIDS recommended Voluntary Medical Male Circumcision in 2007 based on systematic review of observational studies prior to 1999 and three randomized controlled trials (RCTs). To inform updated WHO guidance, we conducted a systematic review and meta‐analysis of impact of circumcision on the risk of HIV infection among heterosexual men.

**Methods:**

Studies in PubMed of HIV incidence and changes in prevalence in heterosexual men by circumcision status were identified. Pooled incidence rate ratios were computed using fixed‐ and random‐effects meta‐analysis and risk of bias was assessed using the ROBINS‐I tool.

**Results and Discussion:**

In three RCTs, the pooled incidence ratio was 0.41 (95% CI 0.30 to 0.56), with risk difference 10 (8 to 12) fewer infections per 1000 person‐years (py). Pooled incidence ratios were 0.34 (0.24 to 0.49) in two post‐RCT follow‐up studies, 0.29 (0.19 to 0.43) in men at high HIV risk (five cohorts), 0.48 (0.33 to 0.70) in four community‐based cohorts before circumcision scale‐up, and 0.56 (0.49 to 0.64) (7 [6 to 8] fewer per 1000 py) in six community‐based cohorts during circumcision and antiretroviral treatment scale‐up. Heterogeneity between studies was low except in men at high HIV risk. We estimated 520,000 (425,000 to 605,000) fewer infections occurred in men by end of 2018 from 22.7 million circumcisions performed since 2008 and increasing by 155,000 (125,000 to 180,000) annually if epidemic conditions remain similar. After exclusion of studies with high risk of bias and those conducted outside Africa, pooled incidence ratios were similar. There was no evidence of confounding nor changes in risk behaviour following circumcision. In post‐hoc exploratory analyses we observed a trend of decreasing effectiveness of circumcision in cohorts with lower HIV incidence.

**Conclusions:**

Efficacy of medical male circumcision on HIV incidence from randomized controlled trials was supported by effectiveness from observational studies in populations with diverse HIV risk and changing epidemic contexts. Voluntary Medical Male Circumcision remains an important evidence‐based intervention for control of generalized HIV epidemics.

## 1 Introduction

Data suggesting a potential link between male circumcision and HIV infection at the individual and population levels were published in 1988 [1] and 1989 [2]. A meta‐analysis of observational studies of HIV prevalence and incidence in circumcised and uncircumcised men reported an adjusted relative risk of HIV infection of 0.42 (0.34 to 0.54) in circumcised compared with uncircumcised men [3]. These results strongly suggested a direct relationship between circumcision and reduced risk of HIV infection, but unmeasured or residual confounding could not be excluded.

Based on these observational data, three randomized controlled trials (RCTs) were launched in 2002 in Kenya, South Africa and Uganda, each enrolling HIV‐negative men consenting to be randomized to immediate or delayed circumcision and followed over 21 to 24 months [4, 5, 6]. The three trials showed 60% lower HIV incidence in circumcised compared with uncircumcised men [7] with no evidence of a delay in protection beyond the recommended 6‐week post‐circumcision abstinence. Informed by this new evidence and models projecting substantial impact, WHO and UNAIDS recommended in 2007 that male circumcision be recognized as an efficacious intervention for HIV prevention and urged rapid implementation of programmes offering circumcision in high HIV incidence heterosexual populations with low rates of circumcision [8].

By end of 2018, an estimated total of 23 million men had been circumcised through Voluntary Medical Male Circumcision (VMMC) programmes implemented in 15 priority countries [9, 10 Fig 3.17 p. 65]. During this same period, the total HIV incidence in eastern and southern Africa decreased by 36% [10 Fig 10.2 p. 188] and access to antiretroviral treatment (ART) increased substantially. To inform updated WHO guidance on VMMC programmes for HIV prevention, we conducted a systematic review of HIV incidence in heterosexual men by circumcision status (Population: HIV‐negative men [excluding cohorts of men who predominantly or exclusively had sex with men], Intervention: circumcision or circumcised, Control: no circumcision or not circumcised, Outcome: HIV infection) in varying epidemic contexts, including men in high HIV incidence cohorts, community‐based cohorts with stable circumcision prevalence, and community‐based cohorts during VMMC scale‐up. For completeness, we included the results from the RCTs and the post‐RCT follow‐up studies on HIV incidence by circumcision status.

## 2 Methods

### 2.1 Literature search

We searched PubMed on 8 January 2019 for articles published since 1999 (year of search in first systematic review of circumcision and HIV [3]) with Medical Subject Headings (MeSH) “HIV Infections/prevention & control” or “HIV Infections/epidemiology” combined with “Circumcision, Male” but excluded articles with Publication Types “comment,” “editorial,” “letter,” “news” or “newspaper article.” The titles and abstracts of 771 identified articles were independently reviewed by two authors (TMMF, WA) and discrepancies reconciled by discussion; articles were retained if they contained primary data on HIV incidence in men by circumcision status and/or changes in HIV prevalence in men by circumcision status as the number of circumcised men increased through VMMC interventions. References in reviews were scanned for additional relevant articles and experts contacted regarding articles recently published, in press or in preparation. In addition, we searched relevant conference abstract databases (Conference on Retroviruses and Opportunistic Infections [CROI], International AIDS Conference [IAC] and International AIDS Society Conference on HIV Science [IAS]) for the previous 5 years. Articles were excluded if they referred to men who exclusively or predominantly had sex with men, lacked primary data on HIV incidence or changes in prevalence by circumcision status, or were not in English. In contrast to earlier reviews [3, 11], we excluded studies reporting only HIV prevalence in men by circumcision status at a single time point.

Retained articles were independently reviewed by two authors (TMMF, MKG) and differences reconciled after discussion. Key features of each study were extracted into summary tables as were the primary results. Supplementary information was sought from public databases (e.g. clinical trial registries) and authors were contacted for additional information or clarifications as necessary. Such additional information is documented where relevant in the tables and footnotes. Studies included in the review and meta‐analysis were identified by first author and year of publication and supplemented in figures with ISO‐3166 3‐letter country codes. In March 2019, we attempted to register the review protocol in the PROSPERO database (https://www.crd.york.ac.uk/PROSPERO/) but were refused as we were too far advanced in the process. The PubMed search was updated on 14 January 2020.

### 2.2 Statistical methods

Studies were grouped by design and context according to pre‐specified subgroups (RCTs, post‐RCT follow‐up, men at high risk of infection, and community‐based studies before and during circumcision scale‐up) and presented by approximate date order (midpoint between first enrolment and last follow‐up). Adjusted incidence rate ratios (IRs) were pooled over studies by computing the weighted average of the study‐specific log incidence ratios with weights inversely proportional to variance (fixed‐effects meta‐analysis model) and back‐transformed to the ratio scale. Where an adjusted IR was not available the crude IR was used. Heterogeneity was assessed with Cochran’s Q and the *I* ^2^statistic [12]. To quantify the impact of heterogeneity within study context, we also fitted the DerSimonian and Laird random effects model [13] using Stata v13.1 (StataCorp, College Station, TX, USA).

Absolute differences in incidence (95% confidence intervals [CIs]) attributable to circumcision were computed from the fixed‐effects pooled adjusted IRs (95% CIs) and the mean HIV incidence in uncircumcised men (total HIV infections per total person‐years at risk summed over all relevant studies). For studies not reporting person‐years at risk, we computed time at risk from the number of infections divided by the reported incidence per 100 person‐years (100 py). Where necessary, we estimated incidence per 100 py from the 1‐year cumulative life table incidence expressed in percent. Where cumulative incidence was only reported beyond one year (e.g. Kaplan‐Meier cumulative 2‐ or 6‐year incidence), we estimated an average annual incidence $P_{1}=1-{\left(1-P_{n}\right)}^{\left(1/n\right)}$, where n is the number of years and $P_{j}$ the cumulative incidence after j years.

The risk of bias in the observational studies was assessed using the ROBINS‐I tool for studies of interventions [14] and its adaptation to exposures for prospective studies [15, 16]. Post‐hoc sensitivity analyses were performed excluding studies outside Africa and those assessed as having serious risk of bias. In addition, potential associations between IRs and time period, HIV incidence, circumcision prevalence and ART prevalence in women were assessed graphically and by meta‐regression (Stata command metareg).

The number of HIV infections averted by circumcisions performed by VMMC programmes in 15 priority countries was computed from the cumulative py since circumcision to end of 2018 based on the number of circumcisions performed each calendar year (assumed uniformly within each year) from 2008 to 2018 [9, 10; Fig 3.17, p. 65] and the estimated absolute reduction in HIV incidence attributable to circumcision from community‐based cohorts during circumcision scale‐up.

## 3 Results and Discussion

**Figure 1:**
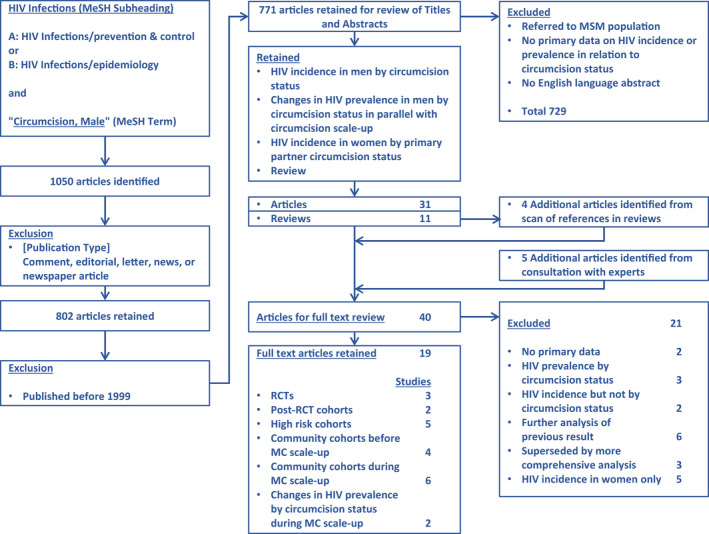
PRISMA flowchart

A total of 31 articles and 11 reviews were retained for full text review (Figure 1). An additional four articles were identified from references in reviews and five from consultation with experts. No new studies or articles were identified from review of conference abstracts. Twenty‐one articles were excluded as they contained no primary data (2), referred only to HIV prevalence by circumcision status (3), did not include HIV incidence stratified by circumcision status (2), were a further analysis of a previous result (6) (Table S1), had been superseded by a more comprehensive analysis (3) (Table S1), or referred exclusively to HIV incidence in women (5). The 19 full text articles retained included 20 studies of HIV incidence in men by circumcision status – three RCTs, two extended follow‐up of former RCT participants, five cohorts of men at high HIV risk, four community‐based cohorts before and six during circumcision scale‐up, and two studies of changes in HIV prevalence in men by circumcision status during circumcision scale‐up. The twenty cohorts included men in Kenya (7), Uganda (6), South Africa (4), seven southern and eastern African countries (1), and India (2) (Table 1).

**Table 1:** Randomised controlled trials and observational studies of circumcision and HIV incidence in men by study type, setting and approximate date order of implementation

Study (first author publication year)	Setting and location	Period	HIV infections [person‐years] (incidence per 100 py)†	Incidence ratio (95% CI)	Notes
A) Randomised controlled trial					
Auvert 2005 [4]	Orange Farm, Gauteng, South Africa	Jul‐2002 – Apr‐2005	Circ. 20 [2354] (0.85) Not circ. 49 [2339] (2.11)	mITT^a^ 0.40 (0.24 to 0.68) ‘As treated’^b^ 0.24 (0.14 to 0.44)	1546 men randomized to immediate and 1582 to delayed circumcision
Bailey 2007 [5]	Nyanza Province, Kenya	Feb‐2002 – Dec‐2006	Circ. 22 [2084^c^] (1.06^d^) Not circ. 47 [2214^c^] (2.12^d^)	mITT 0.41 (0.24 to 0.70) ‘As treated’ 0.40 (0.23 to 0.68)	1391 men randomized to immediate and 1393 to delayed circumcision
Gray 2007 [6]	Rakai District, Uganda	Aug‐2002 – Dec‐2006	Circ. 22 [3352] (0.66) Not circ. 45 [3392] (1.33)	mITT 0.43 (0.24 to 0.75) ‘As treated’ 0.40 (0.23 to 0.70)	2474 men randomized to immediate and 2522 to delayed circumcision
Pooled			Circ. 64 [7791] (0.82) Not circ.141 [7945] (1.77)	mITT 0.41 (0.30 to 0.56)^e^	Absolute difference:^f^ 10 fewer (from 8 to 12 fewer) per 1000 py Heterogeneity statistics: *I* ^2^ = 0.0%, χ^2^_(2)_ = 0.03, *p* = 0.98
B) Extended follow‐up of former RCT participants					
Mehta 2013 [18]	Nyanza Province, Kenya	Feb‐2002 – Sep‐2010	Circ. 47 [5744^c^] (0.82)^g^ Not circ. 79 [4107^c^] (1.92)^g^	Crude 0.38 (0.26‐0.55) Adj. 0.42 (0.26 to 0.66)^h^	Estimated crude IR from 2 to 6 years 0.36 (0.22 to 0.61) computed as weighted difference between crude IR over full 6‐year period and “as treated” IR over 2 years from RCT (0.40 [0.23 to 0.68])[5]^i^
Gray 2012 [19]	Rakai District, Uganda	Dec‐2006 – Dec‐2010	Circ. 48 [9628] (0.50) Not circ. 21 [1087] (1.93)	Crude 0.26 (0.15 to 0.43) Adj. 0.27 (0.16 to 0.45)^j^	
Pooled			Circ. 95 [15,373] (0.62) Not circ.100 [5194] (1.93)	Adj. 0.34 (0.24 to 0.49)^k^	Absolute difference^f^: 13 fewer (from 10 to 15 fewer) per 1000 py Heterogeneity statistics: *I* ^2^ = 35.4%, χ^2^_(1)_ = 1.55, *p* = 0.21
C) Cohorts of men at high risk of HIV infection					
Cameron 1989 [20]	STI clinic patients, Nairobi, Kenya	Mar‐1986 – Dec‐1987	Circ. 6 [60.8^l^] (9.9) Not circ. 18 [20.7^l^] (87.1)	Crude0.11 (0.04 to 0.30)^m^ Adj. 0.12 (0.04 to 0.33)^n^	Circumcision prevalence 214/293 (73%)
Lavreys 1999 [21]	Trucking company employees, Mombasa, Kenya	Mar‐1993 – Jun‐1997	Circ. 32 [1280^o^] (2.5) Not circ. 11 [186^o^] (5.9)	Crude 0.43 (0.23 to 0.91) Adj. 0.25 (0.12 to 0.53)^p^	Circumcision prevalence 651/746 (87%)
Gray 2000 [22]	Serodiscordant couples, Rakai District, Uganda	Nov‐1994 – Oct‐1998	Circ. 0 [106] (0.0) Not circ. 40 [239](16.7)	Crude 0.00 (0.00 to 0.22)^q^ Adj. ‐	Circumcision prevalence 50/187 (27%)
Reynolds 2004 [23]	STI clinic patients, Pune, Maharashtra, India	May‐1993 – 2000	Circ. 2 [285] (0.7) Not circ.165 [3013] (5.5)	Crude 0.13 (0.02 to 0.47) Adj. 0.15 (0.04 to 0.62)^r^	Circumcision prevalence 191/2298 (8%)
Hughes 2012 [24]	Serodiscordant couples, 7 countries southern and eastern Africa^s^	Nov‐2004 – Oct‐2008	Circ. 20 [1712] (1.2) Not circ. 24 [1297] (1.9)	Crude 0.53 (0.29 to 0.96) Adj. 0.53 (0.29 to 0.96)^t^	Circumcision prevalence 1225/2223 (55%). Number of infections and py from [25]. ART use in female partner reported in 543 of 12,966 quarterly visits (4%) [25].
Pooled			Circ. 60 [3444] (1.7) Not circ.258 [4756] (5.4)	Adj. 0.29 (0.19‐0.43)^k^	Absolute difference^f^: 39 fewer (from 31 to 44 fewer) per 1000 py Heterogeneity statistics: *I* ^2^ = 67.0%, χ^2^_(4)_ = 12.1, *p* = 0.017
D) Community‐based cohorts before circumcision scale‐up					
Gray 2000 [22]	Rakai District, Uganda	Nov‐1994 – Oct‐1998	Circ. 18 [1683] (1.07) Not circ.154 [8548] (1.80)	Crude0.59 (0.34 to 0.97)^q^ Adj. 0.53 (0.33 to 0.87)^u^	Proportion circumcised 908/5516 (16%).
Shaffer 2007 [26]	Kericho, Rift Valley Province, Kenya	Jun‐2003 – Dec‐2006	Circ. 17 [2165] (0.79) Not circ. 13 [524] (2.48)	Crude 0.31 (0.15 to 0.64) Adj. 0.34 (0.16 to 0.73)^v^	Proportion circumcised 1108/1378 (80%)
Kim 2016[27]	Kenya (national)	Aug‐2006 – Dec‐2007	Circ. 23 [5618^w^] (0.41) Not circ. 4 [858^w^] (0.47)	Crude 0.88 (0.30 to 2.53)^x^ Adj. Not reported	Cross‐sectional survey – recent HIV infection (approximately last 12 months) using Limiting Antigen Avidity Enzyme Immunoassay (LAg) assay. Proportion circumcised 5618/6476 (87%). National ART prevalence 40% in 2007 [28].
Dandona 2013 [29]	Guntur District, Andhra Pradesh State, India	2004 – Jun‐2011	Circ. 1/744^y^ (‐) Not circ. 22/3265^y^ (‐)	Crude 0.20 (0.03 to 1.47)^z^ Adj. 0.07 (0.01 to 0.83)^aa^	Proportion circumcised 744/4009 (19%)
Pooled			Circ. 58 [9466] (0.61)^bb^ Not circ. 171 [9930] (1.72)^bb^	Adj. 0.48 (0.33 to 0.70)^k^	Absolute difference^f^: 9 fewer (from 5 to 12 fewer) per 1000 py Heterogeneity statistics: *I* ^2^ = 33.9%, χ^2^_(3)_ = 4.54, *p* = 0.21
E) Community‐based cohorts during circumcision scale‐up					
Grabowski 2017 [30]	Rakai District, Uganda	Apr‐1999 – Aug‐2016	Circ. 97 [16,256] (0.60) Not circ.283 [25,893] (1.09)	Crude 0.54 (0.43 to 0.69) Adj. 0.62 (0.48 to 0.79)^cc^	Circumcision prevalence increased from 15% in 1999 to 59% in 2016 and ART coverage from 12% to 68% among HIV positive adults from 2006 to 2016 (from 8% to 61% in men and from 13% to 72% in women)
Lissouba 2011 [31]	Orange Farm, Gauteng, South Africa	Oct‐2007 – Apr‐2008	Circ. 6 [273^o^] (2.2) Not circ. 37 [661^o^] (5.6)	Crude 0.40 (0.16 to 0.98) Adj. 0.35 (0.14 to 0.89)^dd^	Baseline survey. Recent HIV infection (last 15 months) using BED assay in men ages 22 to 34 yr using BED assay. Proportion circumcised (clinical examination) 199/698 (29%).Estimated 16% national ART coverage in women in 2007 [32].
Auvert 2013 [33]	Orange Farm, Gauteng, South Africa	Oct‐2010 – Jun‐2011	Circ. ‐ [‐] (1.2) Not circ.‐ [‐] (3.9)	Crude 0.43 (0.25 to 0.69)^ee^ Adj. 0.41 (0.23 to 0.70)^ff^	Follow‐up survey. Recent HIV infection (last 12 months) using BED assay (using cut‐off 1.51, correction‐1, corresponding to an approximate 12‐month window for new infections; alternative parameters with different assay windows gave comparable results [see Table 5 in Auvert 2013]). Proportion circumcised 1771/3338 (53%). Estimated 46% national ART coverage in women in 2009 [32].
Vandormael 2019 [34]	Hlabisa, KwaZulu‐Natal, South Africa	2009 – 2017	Circ. 89^gg^ [7840^gg^] (1.14) Not circ.429^gg^[16,932^gg^] (2.54)	Crude 0.45 (0.35 to 0.57)^q^ Adj. 0.58 (0.47 to 0.71)^hh^	From 2009 to 2016 circumcision prevalence increased from 3% to 33% and ART coverage from 18% to 37% in men and 19% to 49% in women.
Borgdorff 2018 [35]	Siaya County, Nyanza Province, Kenya	Oct‐2010 – Sep‐2016	Circ. 18 [3936^ii^] (0.46) Not circ. 73 [10,459^ii^] (0.70)	Crude 0.66 (0.37 to 1.11)^q^ Adj. Not reported	Proportion circumcised 1211/4429 (27%). ART prevalence in women 126/296 (43%). Person‐years of follow‐up estimated assuming average follow‐up time was the same for circumcised and uncircumcised men and corresponded to the mean follow‐up for all men in the cohort. No information on circumcisions performed during study period. Indirect evidence from Kenya Demographic and Health Survey 2014 reported circumcision prevalence in Nyanza Province increased from 45% in 2008 to 72% in 2014 [36].
Kagaayi 2019 [37]	Fishing Communities, Lake Victoria, Uganda	Nov‐2011– Feb‐2017	Circ. 53 [3635] (1.46) Not circ. 69 [1926] (3.58)	Crude 0.41 (0.28 to 0.59)^q^ Adj. 0.46 (0.32 to 0.67)^jj^	Circumcision prevalence increased from 35% to 65% and ART coverage from 16% to 82% among HIV‐positive adults from 2011 to 2016 (13% to 78% in men and 18% to 85% in women).
Pooled			Circ. 216 [28,233] (0.77)^kk^ Not circ.953 [61,553] (1.55)^kk^	Adj. 0.56 (0.49 to 0.64)^k^	Absolute difference^f^: 7 fewer (from 6 to 8 fewer) per 1000 py Heterogeneity statistics: *I* ^2^ = 0.0%, χ^2^_(5)_= 4.35, *p* = 0.50

^†^ ‘‐’, Not reported; ‘Circ.’, Circumcised; ‘Not circ.’, Not circumcised.

^a^ Modified intention‐to‐treat analysis, excluding men subsequently identified as HIV‐positive at enrolment

^b^ Analysis according to actual circumcision status

^c^ Computed from estimated annualized incidence and number of infections

^d^ Annualized incidence estimated from reported cumulative 2‐year life table incidence [*P*_1_ = 1 ‐ (1 – *P*_2_)^(1/2)^]

^e^ Weighted average of log IRs with weights inversely proportional to variance back‐transformed to ratio scale

^f^ Computed from pooled IR, confidence interval and incidence in uncircumcised men

^g^ Annualized incidence estimated from reported cumulative 6‐year life table incidence [*P*_1_ = 1 ‐ (1 ‐ *P*_6_)^(1/6)^]

^h^ Adjusted for baseline age and time‐varying sexual behaviours and sexually transmitted infections

^i^ Computed as weighted difference of log IRs with weights inversely proportional to variance back‐transformed to ratio scale

^j^ Adjusted for sociodemographic characteristics and time‐varying sexual behaviours

^k^ Weighted average of log adjusted IRs with weights inversely proportional to variance back‐transformed to ratio scale

^l^ Computed from mean follow‐up (weeks) by seroconversion and circumcision status

^m^ Computed from number of infections and py at risk

^n^ Odds ratio, adjusted for frequency of contact with prostitutes (once, two or more) and genital ulcer disease

^o^ Computed from reported incidence and number of infections

^p^ Adjusted for occupation (driver or assistant vs. mechanic or ancillary worker), religion, frequency of extramarital sex

^q^ Computed from number of infections and py at risk (exact confidence interval)

^r^ Adjusted for fixed (religion, education, living with family) and time‐varying (calendar year, age, marital status, multiple sex partners, sex‐worker partners, condom use, tattoos, medical injections) risk factors

^s^ Botswana, Kenya, Rwanda, South Africa, Tanzania, Uganda, Zambia

^t^ Adjusted for fixed (age and HSV‐2 status at enrolment) and time‐varying (genital ulcer disease, and partner plasma viral load and condom use) risk factors

^u^ Adjusted for randomisation arm, age, marital status, extramarital sex partners, sexually transmitted infection diagnoses

^v^ Adjusted for age, education and religion

^w^ Number of recent infections from published paper Tables 1 and 2 reversing labels ‘Circumcised’ and ‘Not circumcised’ which had been switched in error [personal communication Andrea Kim, December 2018]. Person‐years computed assuming 12‐month window for recent infection

^x^ Computed from number of infections and py at risk (approximate confidence interval from log IR)

^y^ Number of HIV infections/number at risk (incidence not reported)

^z^ Odds ratio computed from number of infections and number at risk

^aa^ Odds of HIV infection adjusted for standard of living, occupation, spouse HIV status, lifetime sex partners, and condom use in past 6 months

^bb^ Number of HIV infections and py from African studies only

^cc^ Adjusted for survey round, 5‐year age groups, education, sex partners in past year, sex with partners outside community, self‐reported genital ulcer disease in past year, self‐reported non‐marital partnership and consistent condom use, type of community (agrarian, trading), and community HIV prevalence

^dd^ Adjusted for age, ethnic group, marital status, lifetime sexual partners, sexual partners in past year, consistent condom use with non‐spousal partners and HSV‐2 status

^ee^ Weighted by circumcision propensity score from logistic regression on age, ethnic group, religion, ever fathered child, occupation, age at first intercourse, alcohol consumption, education level, and ever married

^ff^ Additionally adjusted for age, lifetime sexual partners, and number of and consistent condom use with non‐spousal partners in the last 12 months

^gg^ Personal communication Alain Vandormael January 2020

^hh^ Adjusted for age, marital status, household income, condom use, out‐migration, female HIV prevalence and female ART coverage

^ii^ Estimated from number at risk and mean follow‐up per man (3.25 py)

^jj^ Adjusted for survey round, age, marital status, education and sexual behaviour (sex partners, sex with partners outside community and self‐reported genital ulcer disease in past year, sex and condom use with non‐stable partners)

^kk^ Number of HIV infections and py excluding one study for which information not presented (Auvert 2013 [33])

### 3.1 Randomized controlled trials

**Figure 2:**
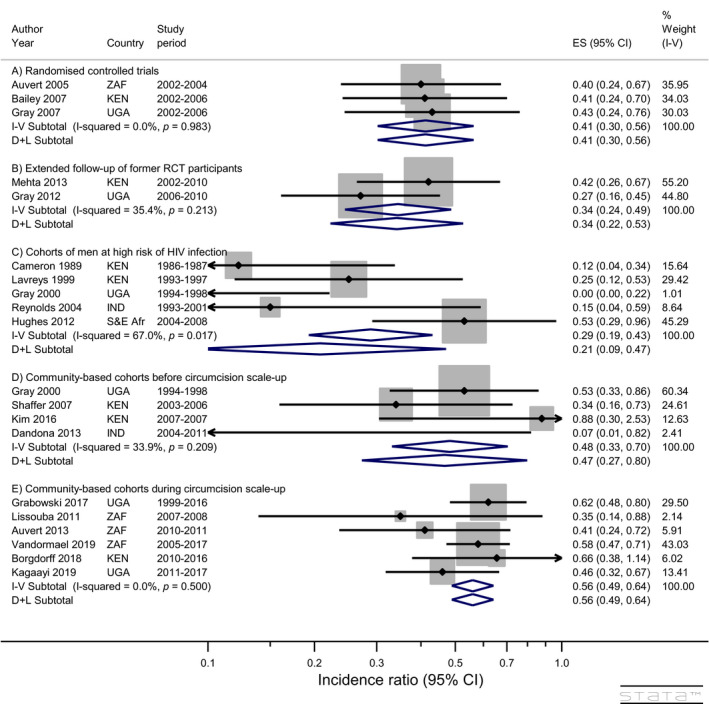
Impact of circumcision on HIV incidence in men (all studies). Note: “S&E Afr” = Seven countries in southern and eastern Africa (BWA, KEN, RWA, TZA, UGA, ZAF, ZMB)

The three RCTs [4, 5, 6] followed a similar design. Consenting volunteers were randomized to immediate or delayed circumcision and followed for up to 21 or 24 months. Follow‐up visits included HIV testing and interview on recent sexual behaviours and other factors potentially associated with risk of HIV infection. Not all men had completed their final follow‐up visit when each trial was stopped due to efficacy at interim analysis. All control arm participants were offered circumcision after trial closure. The estimated IRs from the modified intention‐to‐treat analysis (i.e. men as randomised but excluding those subsequently found to have been HIV‐positive at enrolment after retesting stored baseline sera) in the three trials were 0.40 (0.24 to 0.68), 0.41 (0.24 to 0.70) and 0.43 (0.24 to 0.75) respectively with pooled IR 0.41 (0.30 to 0.56) (heterogeneity statistics: *I* ^2^ = 0%, χ^2^_(2)_ = 0.03, *p* = 0.983) (Table 1A and Figure 2A). The average control arm incidence was 1.77 per 100 py (total of 141 infections in 7945 py) with estimated absolute difference in HIV incidence 10 (8 to 12) fewer infections per 1000 py. A secondary “as‐treated” analysis of the RCTs whereby the few trial crossovers (men randomised to immediate circumcision who declined circumcision or chose to be circumcised later and those randomised to delayed circumcision who underwent circumcision before the scheduled end of the study) were analysed according to actual circumcision status gave somewhat lower IR (Table 1A). The pooled “as treated” IR was 0.34 (0.25 to 0.47) and the estimated absolute reduction in risk was 12 (9 to 13) fewer infections per 1000 py. This pooled IR may be closer to the true effect of circumcision on HIV risk but may be affected by unmeasured confounding.

The three RCTs provided clear evidence of a direct effect of circumcision on reduced risk of HIV acquisition suggesting that previous observational study results were not attributable to confounding. However, the studies were not without limitation [7] including stopping before completion of follow‐up because of strong evidence of benefit potentially leading to an overestimate of impact [17].

### 3.2 Extended follow‐up of former RCT participants

Men still under follow‐up in the Kenya RCT at study closure were invited to participate in a follow‐up study with scheduled visits every six months. 1545 (89%) of 1740 eligible men consented, 778 (50%) of whom were initially in the RCT control arm [18]. Approximately half these control arm men chose to be circumcised during follow‐up. The IR was 0.42 (0.26 to 0.66) after adjustment (Table 1B). There was a tendency for men at lower HIV risk to choose circumcision during the extended follow‐up, but no indication that the lower risk of infection in circumcised men was attenuated over 6‐years follow‐up.

The Rakai study followed former trial participants at six‐monthly intervals and offered circumcision to uncircumcised men after study closure. In total, 3566 men contributed 10,716 py of follow‐up [19]. HIV incidence in the post‐trial period was 0.50 per 100 py in circumcised and 1.93 in uncircumcised men with an adjusted IR of 0.27 (0.16 to 0.45). There were no differences in sexual behaviours between men who had been circumcised during the trial, those who were circumcised by the first post‐trial visit and those who remained uncircumcised at that visit. There was no suggestion that the lower risk of infection in circumcised men was attenuated over the 3.5‐year post‐trial follow‐up period. The pooled adjusted risk estimates from the two post‐RCT follow‐up studies was 0.34 (0.24 to 0.49) (Figure 2B), corresponding to an absolute reduction of 13 (10 to 15) fewer infections per 1000 py.

### 3.3 Cohorts of men at high risk of HIV infection

Five cohorts among groups of men at high risk of HIV infection were identified (Table 1C), the first in men presenting to a sexually transmitted infection (STI) clinic in Nairobi, Kenya between 1986 and 1987 who reported recent sexual exposure to a documented cohort of sex workers, 85% of whom were HIV‐positive [20]. The men were tested for HIV at first presentation, treated for STIs and followed for incident HIV infection every 2 weeks for the first three months and then monthly to a maximum of 18 months follow‐up. The HIV incidence was 29 per 100 py in 293 HIV‐negative men with at least one repeat HIV test, 73% of whom were circumcised. The incidence was 10 per 100 py in circumcised and 87 per 100 py in uncircumcised men, with IR 0.12 (0.04 to 0.33) after adjustment for frequency of sexual contacts with sex workers and presence of genital ulcer disease.

A study of trucking company employees in Mombasa, Kenya followed men every 3 months over the period 1993 to 1997 with few losses to follow‐up [21]. There were 32 infections in 651 circumcised men (5%) and 11 in 95 uncircumcised men (12%). The adjusted incidence ratio was 0.25 (0.12 to 0.53).

A subset of men in the Rakai STD Control for AIDS Prevention Study were linked to their wife or primary partner resulting in identification of 187 HIV serodiscordant couples with an HIV‐negative male (50 circumcised and 137 uncircumcised)and positive female partner [22]. There were no new HIV infections in circumcised men during 106 person‐years of follow‐up and 40 in uncircumcised men during 239 py – incidence ratio 0.0 (0.0 to 0.22).

A study of men attending sexually transmitted disease clinics in India in 1993 to 2000 followed 191 circumcised and 2107 uncircumcised men at 3‐monthly intervals for a mean of one year [23]. Two infections occurred in circumcised men (1%) compared with 165 in uncircumcised men (8%). The adjusted incidence rate ratio was 0.15 (0.04 to 0.62).

A further cohort of serodiscordant couples with HIV‐negative male partner was assembled for a RCT of herpes simplex virus type‐2 suppressive therapy and HIV prevention in 14 sites in seven countries in southern and eastern Africa between 2004 and 2007 and followed monthly for 2 years [24, 25]. The cohort consisted of 1225 couples with circumcised and 998 with uncircumcised men. Twenty HIV infections occurred during 1712 py follow‐up of circumcised men (incidence 1.2 per 100 py) and 24 during 1297 person years follow‐up of uncircumcised men (incidence 1.9 per 100 py). The crude incidence ratio was 0.53 (0.29 to 0.96) and was unchanged after adjustment for fixed and time‐varying risk factors.

The pooled adjusted IR (fixed‐effects model) over the five cohorts of men at high risk of HIV infection was 0.29 (0.19 to 0.43) with an estimated risk difference of 39 fewer (31 to 44 fewer) infections per 1000 py. There was high heterogeneity between the studies (*i* ^2^ = 67%) and the random effects meta‐analysis model resulted in a somewhat lower IR and wider confidence interval (0.21 [0.09 to 0.47]; Figure 2C).

### 3.4 Community‐based cohorts before circumcision scale‐up

Four prospective community‐based studies compared HIV incidence in circumcised and uncircumcised men before expansion of circumcision programmes (Table 1D).

A cluster randomized trial to assess the impact of periodic mass STI treatment on HIV incidence was conducted in Rakai District, Uganda between 1994 and 1998 with follow‐ups at 10‐month intervals. Among 5516 HIV‐negative men with a follow‐up HIV test, 908 (16%) were circumcised [22]. HIV incidence was 1.07 and 1.80 per 100 py in circumcised and uncircumcised men respectively with an adjusted incidence ratio of 0.53 (0.33 to 0.87). The subset of men with a documented HIV‐positive partner included in the cohort of men with high risk of infection in Table 1C above represented 23% of HIV infections and 3% of follow‐up in this cohort.

The Kericho HIV Cohort Study followed tea plantation workers in Kenya at six‐monthly intervals from 2003 until 2006. A total of 30 HIV infections occurred in the subset of 1378 HIV‐negative men, 1108 (80%) of whom were circumcised [26]. HIV incidence was 0.79 and 2.48 per 100 py in circumcised and uncircumcised men respectively – adjusted incidence ratio 0.34 (0.16 to 0.73).

A cross‐sectional nationally representative Kenya AIDS Indicator Survey was conducted among residents ages 15 to 64 years in 2007 and included a serological assessment of HIV prevalence. Stored HIV‐positive specimens were subsequently tested for evidence of recent infection using the Limiting Antigen Avidity Enzyme Immunoassay (LAg) assay and separately for evidence of antiretroviral drug use allowing the authors to distinguish long‐standing and recent HIV infections presumed to have occurred within approximately the past 12 months. There were 23 recent HIV infections in 5595 circumcised and 4 in 854 uncircumcised men at risk leading to a crude recency (or incidence) ratio of 0.88 (0.30 to 2.53) under the assumption that any misclassification of recent infection was similar in circumcised and uncircumcised men [27].

The only population‐based cohort study conducted outside Africa was in Andhra Pradesh State, India with a baseline household survey in 2004 to 2005 and follow‐up survey 2010 to 2011 linked on name, address, age, religion and caste [29]. In total, 4009 HIV‐negative men were included in the HIV incidence cohort, 744 (19%) of whom reported being circumcised. One circumcised and 22 uncircumcised men were HIV positive at follow‐up with adjusted odds ratio 0.07 (0.01 to 0.83). The overall HIV incidence in men in the cohort was 1.25 per 1000 py, approximately 10‐fold lower than the incidence in African settings. With such a low incidence the odds ratio is a close approximation to the incidence ratio.

The pooled IR from the four community‐based cohorts without change in circumcision prevalence was 0.48 (0.33 to 0.70), corresponding to an absolute difference of 9 (5 to 12) fewer HIV infections per 1000 py when applied to the incidence in uncircumcised men from the Uganda and two Kenya studies.

### 3.5 Community‐based cohorts during circumcision scale‐up

The remaining six community‐based studies were conducted in Kenya (1), Uganda (2) and South Africa (3) during periods when VMMC programmes were being implemented resulting in substantial increases in circumcision prevalence (Table 1E).

An analysis of 12 survey rounds of the Rakai Community Cohort Study covered the period 1999 to 2016 when circumcision prevalence increased from 17% before 2005 to 60% in 2016 and ART from 12% in 2006 to 68% in 2016 (from 8% to 61% in men and from 13% to 72% in women) [30]. Overall, HIV incidence declined from 1.17 per 100 py before the combination HIV prevention programme to 0.66 in 2016 (44% decrease), from 1.17 to 0.84 (28% decrease) in women, from 1.21 to 0.65 (46% decrease) in uncircumcised men, and from 0.95 to 0.33 (65% decrease) in circumcised men. These decreases were attributed to the combination of ART and circumcision scale‐up, delayed sexual debut in adolescents and a reduction in young men reporting multiple sexual partners. Over the whole study period average HIV incidence was 0.60 and 1.09 per 100 py in circumcised and uncircumcised men respectively with an adjusted incidence ratio 0.62 (0.48 to 0.79).

Following closure of the Orange Farm RCT, the investigators implemented two cross‐sectional studies to capture changes in circumcision coverage and sexual behaviours as circumcision was made available to former control arm participants and other eligible males in the community. The baseline survey in 2007 to 2008 included a clinical examination and blood test to assess HIV prevalence and incidence using the BED assay for recent infection with a 15‐month window [31]. Just under half the men (48%) who reported being circumcised were found not to be circumcised on clinical examination, underlining the importance of clinically determined circumcision status in preference to self‐report. The adjusted incidence rate ratio was 0.35 (0.14 to 0.89) and 29% of men were circumcised.

A second, independent survey in the same community was conducted between 2010 and 2011 by which time circumcision prevalence had increased to 53% [33]. HIV incidence using the BED assay was estimated to be 1.2 and 3.9 per 100 py in circumcised and uncircumcised men respectively. The adjusted incidence rate ratio was 0.41 (0.23 to 0.70).

A demographic surveillance cohort in rural KwaZulu Natal, South Africa was established in 2005 and included annual survey rounds through 2017 [34]. Male circumcision for HIV prevention was introduced from 2009 with prevalence increasing to 33% by 2016. Over the same period, ART coverage increased from 18% to 37% in men and from 19% to 49% in women. The adjusted IR in circumcised compared with uncircumcised men was 0.58 (0.47 to 0.71).

A further demographic surveillance cohort in Siaya County, Nyanza Province, Kenya reported on risk factors for incident HIV infection from the baseline survey in 2010 to 2011 to repeat surveys in 2012 and 2016 [35]. Eighteen infections occurred in 1211 circumcised and 73 in 3218 uncircumcised men. The estimated incidence was 0.46 in circumcised and 0.70 per 100 py in uncircumcised men – crude incidence ratio 0.66 (0.37 to 1.11). No adjusted incidence ratio is available. While there was no information available from the study cohort about changes in circumcision status over the study period, the Kenya National Demographic and Health Surveys reported that circumcision prevalence in Nyanza Province increased from 45% in 2007 to 72% in 2014 [36].

A community‐based cohort was established in four high HIV burden Lake Victoria fishing communities in Rakai and Masaka Districts, Uganda when a combination HIV intervention including VMMC, HIV testing and ART services were introduced from 2011 to 2017 [37]. Circumcision prevalence increased from 35% to 65% and ART coverage from 16% to 82% (13% to 78% in men and 18% to 85% in women). HIV incidence in the cohort declined from 3.43 to 1.59 per 100 py (54% decrease) with similar proportionate declines from 3.63 to 1.86 (49% decrease) in women, from 4.51 to 2.56 (43% decrease) in uncircumcised men, and from 2.08 to 0.96 (54% decrease) in circumcised men. The adjusted incidence ratio in circumcised compared with uncircumcised men was 0.46 (0.32 to 0.67).

The pooled IR from the six community‐based cohorts covering periods of substantial change in circumcision prevalence was 0.56 (0.49 to 0.64), corresponding to an absolute difference of 7 (6 to 8) fewer HIV infections per 1000 py.

### 3.6 Risk of bias

The Risk of Bias assessment for each observational study and domain is detailed in Table S2 and illustrated in Figure S1. All studies had at least moderate risk of bias due to confounding, the best permissible score for observational studies. Adjusted IR estimates were in general very close to the crude estimates suggesting little confounding. Nevertheless, the possibility of unmeasured confounding cannot be entirely excluded. Three studies were assessed as having serious risk of bias due to confounding because adjusted incidence ratios were not reported (Kim 2016 [27], Borgdorff 2018 [35]) or information on risk factors was only collected at a single follow‐up with a long recall interval (Dandona 2013 [29]). The eight studies which assessed circumcision status from clinical examination had low risk of classification bias; the remaining studies relied on self‐reported circumcision status and were assessed as moderate risk. Misclassification due to incorrect self‐report would bias the estimated effect towards the null. One study was considered to have serious risk of bias due to deviations from the intervention as the analysis model did not account for changing circumcision status while the study covered a period when circumcision programmes were active in the study area (Borgdorff 2018 [35]). This study also had high risk of bias due to missing data as only 41% of HIV‐negative men were followed up. Four studies which used cross‐sectional incidence assays to identify recent infections were considered to have moderate risk of bias for assessment of outcome (Kim 2016 [27], Lissouba 2011 [31], Auvert 2013 [33] and Borgdorff 2018 [35]). Such assays are less reliable than repeat serological testing for HIV infection. Non‐differential misclassification of recent infection by circumcision status would bias the estimated effect toward the null. All studies were assessed to have low risk of selective reporting bias (Table S2 and Figure S1) as circumcision was either the primary focus of the incidence study or a pre‐specified factor for adjustment.

### 3.7 Sensitivity analysis

**Figure 3:**
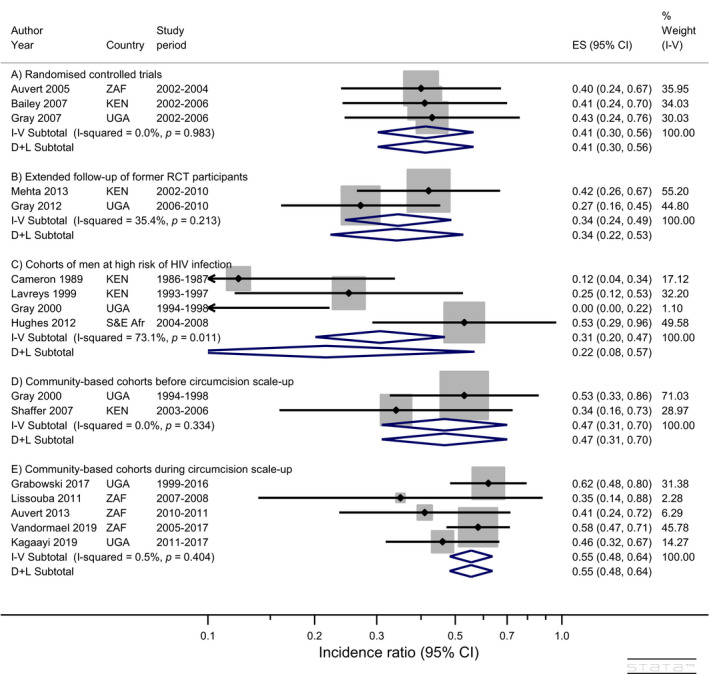
Impact of circumcision on HIV incidence in men (excluding studies with serious risk of bias and those conducted outside Africa). Note: “S&E Afr” = Seven countries in southern and eastern Africa (BWA, KEN, RWA, TZA, UGA, ZAF, ZMB)

After exclusion of the three studies with serious risk of bias and the remaining study conducted in India, the pooled incidence ratios were similar to those calculated from all studies (Figure 3). The study in India [23] contributed 9% weight to the studies in high risk men and IR was the median IR for the five studies. As a consequence, the fixed and random effects pooled estimates changed little and the *i* ^2^ statistic increased from 67% to 73% (Figures 2,3). The pooled IRs and confidence intervals for the community‐based studies before and during circumcision scale‐up were essentially unchanged and the *i* ^2^ statistics were both 0% after exclusions. The estimated absolute reduction in HIV infections for the five remaining community‐based studies during circumcision scale‐up was 8 (6 to 9) fewer infections per 1000 py.

In exploratory analyses of the estimated IRs in the community‐based studies during circumcision scale‐up excluding the study with serious risk of bias we observed no association with time period (mid‐point between first enrolment and last follow‐up), average circumcision prevalence (mean of prevalence in first and last years of the cohort period) or average prevalence of ART coverage in women (mean of prevalence in first and last years of the cohort period) (Figure S2A, C and D). However, IRs monotonically decreased with increasing HIV incidence (Figure S2B). This pattern was also observed in the cohorts of men at high risk of HIV infection and the community‐based studies before circumcision scale‐up (Figure S3A) and when all eleven cohort studies were ordered by incidence (Figure S3B). The trend was significant on a meta‐regression analysis on log HIV incidence (*p* = 0.005). The trend implies that effectiveness of circumcision (1 – incidence ratio) was greatest in settings where HIV incidence was high and lowest in settings where incidence was low.

### 3.8 Estimated infections averted by VMMC programmes

Applying the estimated number of infections averted in the community‐based studies during VMMC and ART scale‐up to the 22.7 million circumcisions performed from 2008 to end 2018 (total 76.4 million post‐circumcision py) we estimated 520,000 (425,000 to 605,000) fewer infections occurred in men by end of 2018 as a direct result of the VMMC programmes in the 15 priority countries. Assuming epidemic conditions remain similar, the number of infections averted in men will increase by 155,000 (125,000 to 180,000) annually by the circumcisions performed to end 2018. These estimates assume that average HIV incidence in the community‐based studies during VMMC scale‐up applies to all 15 priority countries, but do not include secondary and higher order infections averted in women, infants or other men. The corresponding estimated impact after exclusion of studies with high risk of bias was 585,000 (480,000 to 680,000) infections averted by end of 2018, increasing by 175,000 (140,000 to 200,000) each year.

### 3.9 Repeat assessment of HIV prevalence by circumcision status

Two of the six studies which covered periods of circumcision scale‐up also provided information on the HIV prevalence in circumcised and non‐circumcised men at multiple time points in the same community which allows an assessment of how the prevalence ratio evolved (Table 2). Two cross‐sectional population‐based surveys were conducted in the Orange Farm community, the first in 2007 to 2008 including 1988 men and the second in 2010 to 2011 including 3338 men [33]. Circumcision prevalence increased from 17% to 53% and ART use in men from 13% to 26% over the three years. HIV prevalence among men ages 15 to 49 in 2007 to 2008 was 7.2% in circumcised and 17.2% in uncircumcised men, and 6.6% and 17.2% respectively at the second survey (Table 2). The adjusted prevalence ratio was 0.45 (0.26 to 0.79) at baseline and 0.49 (0.38 to 0.62) at the second survey.

**Table 2:** Observational studies of circumcision and HIV prevalence in men in communities with substantial circumcision scale‐up

Study (first author publication year)	Setting and location	Period	HIV prevalence – % (n/N)^†^	Prevalence ratio (95% CI)	Notes			
Auvert 2013 [33]	Orange Farm, South Africa	Baseline: Oct‐2007 – Apr‐2008 Follow‐up: Oct‐2010 – Jun‐2011	Baseline: Circ. 7.2% (15/208) Not circ. 17.2% (96/558) Follow‐up: Circ. 6.6%(117/1771) Not circ. 18.6%(295/1567) Both survey rounds Circ. 6.7% (132/1979) Not circ. 18.4% (391/2125)	Crude 0.42 (0.25‐0.71)^a^ Adj. 0.45 (0.26‐0.79)^b^ Crude 0.35 (0.29‐0.43)^a^ Adj. 0.49 (0.38‐0.62)^c^ Crude 0.36 (0.30‐0.44) Adj. 0.48 (0.39‐0.60)	Baseline HIV prevalence from subset of 766 men (27% circumcised) ages 22 to 34 years (Lissouba 2011 [33]). Circumcision prevalence in men ages 15‐49 years increased from 17% (329/1988) at baseline to 53% (1771/3338) at follow‐up survey. ART use in HIV‐positive men increased from 13% (38/288) at baseline to 26% (109/412) at follow‐up survey.			
Kagaayi 2019 [37]	Fishing Communities, Lake Victoria, Uganda	Five survey rounds from Dec‐2011 to Dec‐2016	2011 survey: Circ. 25% (173/698) Not circ. 39% (518/1313) 2012 survey: Circ. 25% (218/883) Not circ.40% (499/1243) 2013 survey: Circ. 25% (264/1075) Not circ 41% (402/988) 2015 survey: Circ 24% (304/1249) Not circ.42% (383/905) 2016 survey: Circ. 25% (409/1630) Not circ. 44% (391/894) All survey rounds Circ. 25% (1368/5535) Not circ. 41% (2193/5344)	Crude 0.63 (0.54‐0.73)^a^ Adj. Not reported Crude 0.61 (0.54‐0.70)^a^ Adj. Not reported Crude 0.60 (0.53‐0.69)^a^ Adj. Not reported Crude 0.58 (0.51‐0.65)^a^ Adj. Not reported Crude 0.58 (0.51‐0.64)^a^ Adj. Not reported Crude 0.60 (0.57 to 0.64)^a^ Pooled 0.60 (0.56 to 0.63)^d^ Adj. 0.67 (0.62 to 0.73)^e^	Circumcision and ART prevalence by survey round			
					Survey	Circumcision	ART women	ART men
					2011	35% (698/2011)	18% (164/907)	13% (90/691)
					2012	42% (883/2126)	31% (259/844)	18% (127/717)
					2013	52% (1075/2063)	49% (417/856)	34% (228/666)
					2015	58% (1249/2154)	72% (648/900)	56% (382/687)
					2016	65% (1630/2525)	85% (797/940)	78% (623/799)
Pooled			Circ. 20% (1485/7306) Not circ.36% (2487/6910)	Crude 0.58 (0.55 to 0.61) Adj. 0.65 (0.60 to 0.70)^f^	Absolute difference^g^: 148 fewer (from 134 to 162 fewer) per 1000 men Heterogeneity statistics: *I* ^2^ = 82.3%, χ^2^_(1)_ = 5.65, *p* = 0.017			

^†^ Percent (number with infection/ Number tested)

^a^ Computed from reported number of infections and number tested

^b^ Adjusted for age, ethnic group, marital status, number of lifetime sexual partners, number of sexual partners in the past 12 months, consistent condom use with non‐spousal partners and HSV‐2 status using Poisson regression model

^c^ Adjusted for age group

^d^ Weighted average over all 5 survey rounds. Heterogeneity: *I* ^2^ = 0.0%, χ^2^_(4)_ = 1.45, *p* = 0.83

^e^ Adjusted for demographics (gender, age, marital status, education) and sexual behaviours (number of sexual partners in the previous 12 months, sex with partners outside the community of residence, sex with non‐marital partners, condom use and self‐reported genital ulceration)

^f^ Weighted average of log adjusted prevalence ratios with weights inversely proportional to variance back‐transformed to ratio scale

^g^ Computed from pooled adjusted prevalence ratio, confidence interval and prevalence in uncircumcised men.

The open cohort study conducted among Lake Victoria fishing communities involved five cross‐sectional surveys from 2011 to 2016 [37]. Circumcision increased from 35% to 65% and ART from 18% to 85% among women and from 13% to 78% among men (Table 2). HIV prevalence at each survey remained constant at approximately 25% in circumcised and 40% in uncircumcised men with an adjusted pooled prevalence ratio of 0.67 (0.62 to 0.73) (Table 2).

The combined adjusted prevalence ratio estimate was 0.65 (0.60 to 0.70), though this should be interpreted cautiously due to the large difference between the two studies. The estimated absolute difference in risk – 148 fewer infections (from 134 to 162 fewer) per 1000 men – was dominated by the very high HIV prevalence in the Lake Victoria fishing communities and underscores the large impact of circumcision in high HIV prevalence settings. The studies had low risk of bias in all domains except confounding (moderate) and classification (moderate for the Lake Victoria fishing community study due to self‐reported circumcision status) (Table S2F and Figure S1F).

## 4 CONCLUSIONS

The evidence that circumcision reduces the risk of HIV infection in heterosexual men is strong and consistent from a wide diversity of study designs and settings.

Three RCTs showed a 59% (44% to 70%) reduction in incidence and excluded potential confounding as an explanation for the lower incidence. The protective effect was seen soon after circumcision and persisted up to two years. The two cohorts with extended follow‐up of former RCT participants (Figure 2B) demonstrated that the protective effect persisted up to six years and showed no evidence of risk compensation. Studies in men at high risk of infection (Figure 2C) showed a significant reduction in risk in low [22, 23], moderate [24] and high [20, 21]circumcision prevalence settings but with variability of effect size reflecting their diverse settings. The community‐based cohort studies before circumcision scale‐up (Figure 2D) showed a lower risk of infection in circumcised men in communities with both low [22] and high [26, 27] circumcision prevalence. A similar protective effect was seen in a prospective study in India [29]. Community‐based studies conducted during periods of rapid increase in circumcision prevalence due to VMMC program expansion in parallel with scale‐up of ART [30, 31, 33, 34, 35, 37] also showed a lower risk of infection in circumcised men (Figure 2E). In post‐hoc exploratory analyses, we observed a modest but statistically significant dilution of circumcision effectiveness in settings with lower HIV incidence. This may reflect a hypothesized greater protective effect of circumcision in highly exposed men [38], preferential circumcision in men at lower risk of infection not captured or adjusted for in the individual studies, and/or misclassification of self‐reported circumcision status in the community‐based studies during circumcision scale‐up, as occurred in a study in South Africa [31]. Validation of self‐reported circumcision status by clinical examination in further studies of HIV incidence may clarify the impact of such bias which may be more pronounced in settings where circumcision is being actively promoted (social desirability bias).

The estimated absolute reduction in risk was 10 (8 to 12) fewer infections per 1000 py in the RCTs, 13 (10 to 15) fewer in the post‐RCT follow‐up studies, 9 (5 to 12) fewer in the community‐based studies before VMMC scale‐up and 7 (6 to 8) fewer during circumcision scale‐up. In the high‐risk cohorts, we estimated 39 (31 to 44) fewer infections per 1000 py attributable to circumcision, equivalent to 26 (23 to 32) circumcisions being sufficient to prevent one new infection. This illustrates the large impact of circumcision in men at high HIV risk and the importance of prioritizing such populations for VMMC services.

In contrast to earlier reviews [3, 11], we excluded studies reporting HIV prevalence at a single time point because of the recent rapid increase in ART availability. The two studies of changes in HIV prevalence in circumcised and uncircumcised men showed the prevalence and prevalence ratios remained stable in the presence of substantial increases in circumcision and ART. Since circumcision was being promoted in the two communities, we had expected HIV prevalence to decline over time, possibly with a stable prevalence ratio. Prevalence is a balance between in‐ and out‐migration of HIV‐positive men and between HIV incidence and mortality, and increases with improved survival due to ART. It will therefore not necessarily reflect lower incidence.

To the best of our knowledge, this systematic review includes all published articles on HIV incidence in heterosexual men by circumcision status. We did not review articles published in a language other than English and it is possible that some articles which referred to men who exclusively or predominantly had sex with men may have included some relevant information on heterosexual HIV risk. The information on the impact of circumcision in men at high risk of infection was quite variable but the consistent protective effect suggests that the reasons for the heterogeneity lie in concomitant individual social and medical factors, such as presence of STIs, rather than a different biological impact of circumcision. While some studies had serious limitations and biases, restricting the analysis to the observational studies without serious risk of bias had little impact on the pooled estimates and overall conclusions. Some of the variability between studies was reduced after exclusion of the lower quality studies. Our estimate of the number of infections averted by circumcisions performed since 2008 in VMMC programmes is limited by applying an average impact of circumcision from the published studies during VMMC scale‐up to the total circumcisions performed each year in the priority countries. This crude estimate could be refined if information were available on ages of VMMC clients and HIV incidence in the settings where programs were implemented. We did not formally assess the risk of publication bias but consider this unlikely given the impact of circumcision on HIV prevalence first reviewed in 2000 [3] and the cost and complexity of implementing HIV incidence studies.

In settings where VMMC and ART scale‐up occurred in parallel it is difficult to separate the effects of each intervention on overall reduction in HIV incidence and prevalence. An analysis of HIV incidence in individual communities within the Rakai Community Cohort over the period 1999 to 2013 estimated that each 10% increase in circumcision prevalence was associated with a 13% (7% to 18%) reduction in male HIV incidence, and male incidence was 5% lower (from 19% lower to 13% higher) for each 10% increase in ART coverage in women [39]. By 2016 when ART coverage had increased to 72% in women and 61% in men and circumcision to 59%, a greater reduction in HIV incidence had occurred in men (54% [27% to 71%] reduction) than women (32% [6% to 50%] reduction) though the individual impacts of circumcision and ART coverage could not be separately estimated [30].

An important concern with promoting voluntary medical male circumcision for HIV prevention is that circumcised men know they are at lower risk of acquiring HIV and, as a result, may have more sexual partners and/or be less likely to use condoms. Such risk compensation would diminish the impact of circumcision but to date no evidence of risk compensation in circumcised men has been detected [40, 41, 42, 43, 44]. Male circumcision for HIV prevention in high HIV‐prevalence settings with low circumcision coverage is ultimately cost saving due to treatment costs averted [45, 46, 47]. In contrast to other biomedical HIV‐prevention interventions, VMMC is a one‐time intervention with no recurrent cost or resupply requirements. While treatment is critical for people living with HIV the impact on HIV incidence in four large test‐and‐treat cluster randomized trials was disappointing [48, 49, 50, 51, 52]. Combination prevention including high coverage of medical male circumcision remains an important intervention for HIV control in generalized epidemics (high HIV prevalence in the general population) which only occurred in communities with low circumcision prevalence [53]. Promoting male circumcision to reduce the risk of heterosexual transmission in epidemics where circumcision is already commonly practiced for cultural reasons (e.g. religion and/or social norms) would have limited impact on the HIV epidemic. It is important however for countries that have implemented VMMC programmes to develop mechanisms to maintain high circumcision coverage for new cohorts of young men entering the sexually active age range each year. Promoting medical male circumcision for HIV prevention among men who exclusively or predominantly have sex with men would have limited impact as infection risk is dominated by unprotected receptive anal sex [54].

## Authors’ Contributions

TMMF and JS conceived the review and search strategy. TMMF and WA conducted the literature review. TMMF and MKG performed the Risk of Bias assessments. TMMF, JS, MKG, WA, RHG and RB commented on and contributed to the report and provided critical comments on the interpretation of the review. All authors read and approved the final version for publication. All data extracted from the source literature are included in the report and/or supplementary material.

## Competing Interests

TMMF declares receipt of funds from WHO for reviews of safety and efficacy of circumcision methods and impact of circumcision on HIV infection. All other authors declare no competing interests.

## Abbreviations

ART, antiretroviral treatment; IR, incidence rate ratio; pIR, pooled incidence ratio; RCT, randomized controlled trial; VMMC, voluntary medical male circumcision.

## Supporting information


**Table S1:** Articles with relevant data excluded after full text review
**Table S2:** Risk of bias assessment (observational studies)
**Figure S1:** Risk of bias assessment (observational studies)
**Figure S2:** Incidence ratios in community‐based studies in Africa without serious risk of bias during circumcision scale‐up with studies ordered by A) time period, B) HIV incidence in uncircumcised men, C) average circumcision prevalence during scale‐up, and D) average ART prevalence in women during scale‐up
**Figure S3**: Incidence ratios in observational studies in Africa without serious risk of bias by HIV incidence A) by study type and B) as a single group
