# Supplementary material for: Impact of male circumcision on risk of HIV infection in men in a changing epidemic context – systematic review and meta‐analysis
Source: J Int AIDS Soc. 2020 Jun 18;23(6):e25490. doi: 10.1002/jia2.25490 (PMC7303540; doi:10.1002/jia2.25490)
Supplement: Supplementary file 1 — Table S1: Articles with relevant data excluded after full text review Table S2: Risk of bias assessment (observational studies) Figure S1: Risk of bias assessment (observational studies) Figure S2: Incidence ratios in community‐based studies in Africa without serious risk of bias during circumcision scale‐up with studies ordered by A) time period, B) HIV incidence in uncircumcised men, C) average circumcision prevalence during scale‐up, and D) average ART prevalence in women during scale‐up Figure S3: Incidence ratios in observational studies in Africa without serious risk of bias by HIV incidence A) by study type and B) as a single group [file JIA2-23-e25490-s001.pdf]

# Impact of Male Circumcision on Risk of HIV Infection in Men in a Changing Epidemic Context – Systematic Review and Meta-Analysis

# Supporting Information

Farley TMM, Samuelson J, Grabowski MK, Ameyan W, Gray RH, Baggaley R. Impact of male circumcision on risk of HIV Infection in men in a changing epidemic context – Systematic review and meta-analysis. J Int AIDS Soc. 2020;23:e25490.

## Contents

Table S1: Articles with relevant data excluded after full text review ........ 1

Table S2: Risk of bias assessment (observational studies) ........ 2

Figure S1: Risk of bias assessment (observational studies) ........ 9

Figure S2: Incidence ratios in community-based studies in Africa without serious risk of bias during circumcision scale-up with studies ordered by A) time period, B) HIV incidence in uncircumcised men, C) average circumcision prevalence during scale-up, and D) average ART prevalence in women during scale-up........ 10

Figure S3: Incidence ratios in observational studies in Africa without serious risk of bias by HIV incidence A) by study type and B) as a single group ........ 11

References ........ 12

## Table S1: Articles with relevant data excluded after full text review

| Reason for exclusion | Details |
|---|---|
| Further analysis of previous result | 1) Quinn 2000 [1] further analysis of Gray 2000 [2]<br>2) Baeten 2005 [3] further analysis of Lavreys 1999 [4]<br>3) Gray 2009 [5] further analysis of Gray 2007 [6]<br>4) Mahiane 2009 [7] further analysis of Auvert 2005 [8]<br>5) Shaffer 2010 [9] further analysis of Shaffer 2007 [10]<br>6) Mehta 2012 [11] further analysis of Bailey 2007 [12] |
| Superseded by more comprehensive analysis | 1) Santelli 2015 [13] superseded by Grabowski 2017 [14]<br>2) Kong 2016 [15] superseded by Grabowski 2017 [14]<br>3) Tomita 2017 [16] superseded by Vandormael 2019 [17] |

Table S2: Risk of bias assessment (observational studies)

| First author publication year | 1) Confounding | 2) Selection of participants into study | 3) Classification of intervention or exposure | 4) Deviations from intended intervention or exposure | 5) Missing data | 6) Measurement of outcomes | 7) Selection of reported result |
|---|---|---|---|---|---|---|---|
| B) Extended follow-up of former RCT participants | | | | | | | |
| Mehta 2013 [18] | Moderate<br>Self-selection to circumcision in control arm after closure of RCT. Adequate control of confounding. | Low<br>Well-defined cohort from RCT. 195 of 1740 (11%) eligible former trial participants did not consent for extended post-trial follow-up. | Low<br>Documented circumcision procedure in study facilities | Low<br>Circumcision provided in study facilities. Actual date of circumcision used as time-varying covariate. | Low<br>Ongoing cohort follow-up, censored at last known HIV negative test | Low<br>Objective laboratory-based outcome (repeat serology) | Low<br>Pre-defined primary endpoint on full study cohort |
| Gray 2012 [19] | Moderate<br>Self-selection to circumcision after closure of RCT. Adequate control of confounding. | Low<br>Well-defined cohort from RCT control arm. 388/2522 (15%) of former control arm trial participants did not attend first post-trial follow-up. | Low<br>Documented circumcision procedure in study facilities | Low<br>Circumcision provided in study facilities. Actual date of circumcision used as time-varying covariate. | Low<br>Ongoing cohort follow-up, censored at last known HIV negative test | Low<br>Objective laboratory-based outcome (repeat serology) | Low<br>Pre-defined primary endpoint on full study cohort |
| C) Cohorts of men at high risk of HIV infection | | | | | | | |
| Cameron 1989 [20] | Moderate<br>Adjustment for genital ulcer disease and frequency of prostitute contact had limited impact on estimated OR. Residual confounding cannot be excluded. | Low<br>STI patients with self-reported recent contact with prostitute cohort (~ 85% HIV seroprevalence) confirmed during three separate interviews and without reference to circumcision status | Low<br>Circumcision status assessed by clinical examination | Low<br>No new circumcisions during follow-up period. Circumcision cannot be reversed. | Low<br>Similar follow-up in circumcised and non-circumcised men | Low<br>Objective laboratory-based outcome (repeat serology) | Low<br>Circumcision a priori risk factor from previous case-control study in STI patients from same setting |

| First author publication year | 1) Confounding | 2) Selection of participants into study | 3) Classification of intervention or exposure | 4) Deviations from intended intervention or exposure | 5) Missing data | 6) Measurement of outcomes | 7) Selection of reported result |
|---|---|---|---|---|---|---|---|
| Lavreys 1999 [4] | Moderate<br>Adjustment for major HIV risk factors increased magnitude of protective effect of circumcision. Further adjustment for incident sexually transmitted infections had little impact. Potential for unmeasured confounding cannot be excluded. | Low<br>Trucking company employees enrolled without reference to circumcision status. 19% did not return for follow-up visits, not related to HIV risk factors. | Low<br>Circumcision status assessed by clinical examination | Low<br>No new circumcisions during follow-up period. Circumcision cannot be reversed. | Low<br>Similar follow-up rates in circumcised and non-circumcised men | Low<br>Objective laboratory-based outcome (repeat serology) | Low<br>Pre-defined endpoint |
| Gray 2000 [2] | Moderate<br>No adjustment possible | Low<br>Secondary analysis of cohort in cluster-randomized trial of STD intervention. No bias expected between circumcision status and cohort enrolment. | Moderate<br>Self-reported circumcision status | Low<br>Majority of circumcisions performed under age 12 y for traditional or religious reasons. No reported circumcisions during follow-up and none expected in this community. Circumcision cannot be reversed. | Moderate<br>Approx. 75% follow-up rate overall. No information on differences according to HIV risk profile or circumcision status. | Low<br>Objective laboratory-based outcome (repeat serology) | Low<br>Further analysis of cluster randomized trial cohort on a risk factor of major interest from previous studies |
| Reynolds 2004 [21] | Moderate<br>Limited impact of adjustment for major HIV risk factors. Potential for unmeasured confounding cannot be excluded. | Low<br>Condom use in previous 3 months lower in non-enrolees than enrolees in previous analysis of initial cohort enrolled May-1993 – Mar-1995 [22], though condom use during follow-up not associated with circumcision status. | Low<br>Circumcision status assessed by clinical examination | Low<br>No new circumcisions reported during follow-up period. Circumcision cannot be reversed. | Unclear<br>No information on proportion with incomplete outcome nor differences according to HIV risk profile or circumcision status | Low<br>Objective laboratory-based outcome (repeat serology) | Low<br>Further analysis of prospective STI cohort on endpoint of major interest from previous studies |

| First author publication year | 1) Confounding | 2) Selection of participants into study | 3) Classification of intervention or exposure | 4) Deviations from intended intervention or exposure | 5) Missing data | 6) Measurement of outcomes | 7) Selection of reported result |
|---|---|---|---|---|---|---|---|
| Hughes 2012 [23] | Moderate<br>No effect of adjustment for risk factors for HIV infection on estimated impact of circumcision | Low<br>Highly selected group of serodiscordant couples in RCT, but no evidence of any relation to circumcision other than HIV status | Low<br>Circumcision determined by clinical examination | Low<br>Circumcision cannot be reversed | Low<br>Low loss to follow-up rate in RCT | Low<br>Objective laboratory-based outcome (repeat serology) | Low<br>One of 21 factors investigated for potential to modify per-act transmission probability, of primary interest from previous publications and a pre-specified factor for subgroup analysis in the original RCT. |
| D) Community-based cohorts before circumcision scale-up | | | | | | | |
| Gray 2000 [2] | Moderate<br>Limited impact of adjustment for potential sociodemographic and behavioural confounders assessed at baseline and during follow-up | Low<br>Secondary analysis of cohort included in cluster-randomized trial of STD intervention. No bias expected between circumcision status and cohort enrolment. | Moderate<br>Self-reported circumcision status | Low<br>Majority of circumcisions performed under age 12 yr for traditional or religious reasons | Moderate<br>Approx. 75% follow-up rate overall. No information on differences according to HIV risk profile or circumcision status. | Low<br>Objective laboratory-based outcome (repeat serology) | Low<br>Further analysis of cluster randomized trial cohort on a risk factor of major interest from previous studies. |
| Shaffer 2007 [10] | Moderate<br>Limited impact of adjustment for potential sociodemographic and behavioural confounders assessed at baseline on risk estimate | Low<br>Rates of refusal to participate in cohort and information on HIV risk factors between refusers and participants not reported. Not considered likely to be related to circumcision status. | Moderate<br>Self-reported circumcision status | Low<br>Circumcision cannot be reversed. No reported circumcisions during follow-up. 98% of circumcisions performed under age 20 years while mean age of cohort 31 years. | Low<br>10% of cohort missing outcomes | Low<br>Objective laboratory-based outcome (repeat serology) | Low<br>Risk factor of primary interest given prior publications, and RCT results which motivated the specific analysis |

| First author publication year | 1) Confounding | 2) Selection of participants into study | 3) Classification of intervention or exposure | 4) Deviations from intended intervention or exposure | 5) Missing data | 6) Measurement of outcomes | 7) Selection of reported result |
|---|---|---|---|---|---|---|---|
| Kim 2016 [24] | Serious Single multinomial logistic regression model fitted to incident, prevalent and absence of HIV infection in both men and women, together with an age x circumcision interaction term. Impossible to extract adjusted effect of circumcision on HIV incidence or prevalence and assess impact of adjusting for potential confounding factors. | Low High participation rates (80% blood draw of eligible individuals) and no evidence participation rates related to HIV risk and/or circumcision | Moderate Self-reported circumcision status | Low Circumcision cannot be reversed | Low Few missing data and no reason expected for any association with outcome and exposure | Moderate Incidence estimated from cross-sectional Limiting Antigen Avidity Enzyme Immunoassay ("LAg") assay rather than repeat serology. Sensitivity of estimated incidence ratio to different assumptions on recent infection window not explored. Even if misclassification errors non-differential, impact would bias estimate towards the null. | Low Risk factor of a priori interest |
| Dandona 2013 [25] | Serious Long interval between baseline and follow-up surveys (5-6 years) and long recall period for assessment of behavioural risk factors | Moderate 8,390 of 12,066 (70%) HIV-negative men and women in baseline survey traced and provided follow-up blood for HIV testing. Participation rate 75% for rural and 63% for urban men. | Moderate Self-reported circumcision status | Low Circumcision cannot be reversed | Low Few details provided | Low Objective laboratory-based outcome (repeat serology) | Low One of 16 risk factors investigated for potential impact on HIV incidence, second strongest association after HIV-positive spouse |

| First author publication year | 1) Confounding | 2) Selection of participants into study | 3) Classification of intervention or exposure | 4) Deviations from intended intervention or exposure | 5) Missing data | 6) Measurement of outcomes | 7) Selection of reported result |
|---|---|---|---|---|---|---|---|
| E) Community-based cohorts during circumcision scale-up | | | | | | | |
| Grabowski 2017 [14] | Moderate<br>Major relevant confounding factors assessed and adjusted for, including time varying circumcision status and risk factors. Potential for unmeasured confounding cannot be excluded. | Moderate<br>Participation rates constant over survey rounds, though evidence that younger persons and those with high-risk sexual behaviours more likely to be lost to follow-up than those with low risk behaviours. | Moderate<br>Self-reported circumcision status | Low<br>Circumcision cannot be reversed | Low<br>Younger men more likely to be lost to follow-up, but no evidence of association with circumcision status. Analysis adjusting for age sufficient to control for differential drop-out rates. | Low<br>Objective laboratory-based outcome (repeat serology) | Low<br>Risk factor of a priori interest |
| Lissouba 2011 [26] | Moderate<br>Adjustment for potential sociodemographic and behavioural confounders had only moderate impact on risk estimate. Potential for unmeasured confounding cannot be excluded. | Low<br>Survey response rate 74%. | Low<br>Circumcision status determined by clinical examination | Low<br>Circumcision cannot be reversed | Low | Moderate<br>Potential for misclassification as recent infection assessed using HIV-1 Calypte Incidence BED EIA [BED] assay. Sensitivity of estimated incidence ratio to different assumptions on recent infection window not explored. Even if misclassification errors non-differential, impact would be bias towards the null. | Low<br>Pre-defined primary endpoint and subject of the community intervention |
| Auvert 2013 [27] | Moderate<br>Limited impact of adjustment for major potential confounders, but unmeasured cofounding cannot be excluded | Low<br>Survey response rate 80% | Low<br>Circumcision status assessed by clinical examination | Low<br>Circumcision status assessed by clinical examination | Low | Moderate<br>Potential for misclassification as recent infection assessed using HIV-1 Calypte Incidence BED EIA [BED] assay. Incidence ratio estimates appeared robust to different cutoff values. | Low<br>Predefined primary endpoint |

| First author publication year | 1) Confounding | 2) Selection of participants into study | 3) Classification of intervention or exposure | 4) Deviations from intended intervention or exposure | 5) Missing data | 6) Measurement of outcomes | 7) Selection of reported result |
|---|---|---|---|---|---|---|---|
| Vandormael 2019 [17] | Moderate<br>No assessment of circumcision-specific confounders, but major risk factors included in model; residual confounding cannot be excluded. | Moderate<br>Average 35% of eligible subjects contacted and tested for HIV. Adjustment for out-migration and not having HIV test using propensity-score weighting. | Moderate<br>Self-reported circumcision status | Low<br>Circumcision cannot be reversed | Moderate<br>Average 35% of eligible subjects contacted and tested for HIV | Low<br>Objective laboratory-based outcome (repeat serology) | Low<br>Risk factor of a priori interest from previous studies |
| Borgdorff 2018 [28] | Serious<br>Potential for more men at higher risk of HIV infection to choose circumcision within VMMC program. No data presented on impact of potential confounders. | Low | Moderate<br>Self-reported circumcision status | Serious<br>No information on changes in circumcision status during follow-up period. National VMMC programme initiated in 2008 and likely to have included many men in study cohort. | Serious<br>Only 41% of participants HIV-negative in first survey present in a follow-up cohort | Moderate<br>Home-based HIV testing using established and validated serological testing algorithm. Self-reported HIV infection taken as valid endpoint in absence of HIV test, but self-reported HIV-negative test not considered a valid endpoint. | Low<br>Pre-defined outcome and exposure of specific interest given context of VMMC programme expansion in country following results from RCTs. |
| Kagaayi 2019 [29] | Moderate<br>Limited impact of adjustment for major potential confounders, but unmeasured cofounding cannot be excluded | Low<br>Low refusal rates | Moderate<br>Self-reported circumcision status | Low<br>Circumcision cannot be reversed | Low<br>Low rates of missing data | Low<br>Objective laboratory-based outcome (repeat serology) | Low<br>Exposure of primary interest |
| F) Repeat cross-sectional cohorts (changes in HIV prevalence ratio) | | | | | | | |
| Auvert 2013 [27] | Moderate<br>Limited impact of adjustment for major potential confounders after age differences accounted for | Low<br>Survey response rate 80% | Low<br>Circumcision status assessed by clinical examination | Low<br>Circumcision cannot be reversed | Low | Low<br>Objective laboratory-based outcome (serology) | Low<br>Pre-defined primary endpoint |

| First author publication year | 1) Confounding | 2) Selection of participants into study | 3) Classification of intervention or exposure | 4) Deviations from intended intervention or exposure | 5) Missing data | 6) Measurement of outcomes | 7) Selection of reported result |
|---|---|---|---|---|---|---|---|
| Kagaayi 2019 [29] | Moderate<br>Limited impact of adjustment for major potential confounders | Low<br>Low refusal rates | Moderate<br>Self-reported circumcision status | Low<br>Circumcision cannot be reversed | Low<br>Low rates of missing data | Low<br>Objective laboratory-based outcome (serology) | Low<br>Pre-defined primary endpoint |

## Figure S1: Risk of bias assessment (observational studies)

| Author Year | Country | Study period | 1) Confounding | 2) Selection bias | 3) Classification bias | 4) Deviation from intervention | 5) Missing data | 6) Outcome | 7) Selective reporting |
|---|---|---|---|---|---|---|---|---|---|
| **B) Extended follow-up of RCT participants** | | | | | | | | | |
| Mehta 2013 | KEN | 2002-2010 | Moderate | Low | Low | Low | Low | Low | Low |
| Gray 2012 | UGA | 2006-2010 | Moderate | Low | Low | Low | Low | Low | Low |
| **C) Cohorts of men at high risk of HIV infection** | | | | | | | | | |
| Cameron 1989 | KEN | 1986-1987 | Moderate | Low | Low | Low | Low | Low | Low |
| Lavreys 1999 | KEN | 1993-1997 | Moderate | Low | Low | Low | Low | Low | Low |
| Reynolds 2004 | IND | 1993-2003 | Moderate | Low | Low | Low | No Information | Low | Low |
| Gray 2000 | UGA | 1994-1998 | Moderate | Low | Moderate | Low | Moderate | Low | Low |
| Hughes 2012 | S&E Afr | 2004-2008 | Moderate | Low | Low | Low | Low | Low | Low |
| **D) Community-based cohorts before circumcision scale-up** | | | | | | | | | |
| Gray 2000 | UGA | 1994-1998 | Moderate | Low | Moderate | Low | Moderate | Low | Low |
| Shaffer 2007 | KEN | 2003-2006 | Moderate | Low | Moderate | Low | Low | Low | Low |
| Kim 2016 | KEN | 2007-2007 | Serious | Low | Moderate | Low | Low | Moderate | Low |
| Dandona 2013 | IND | 2004-2011 | Serious | Moderate | Moderate | Low | Low | Low | Low |
| **E) Community-based cohorts during circumcision scale-up** | | | | | | | | | |
| Grabowski 2017 | UGA | 1999-2016 | Moderate | Moderate | Moderate | Low | Low | Low | Low |
| Lissouba 2011 | ZAF | 2007-2008 | Moderate | Low | Low | Low | Low | Moderate | Low |
| Auvert 2013 | ZAF | 2007-2011 | Moderate | Low | Low | Low | Low | Moderate | Low |
| Vandormael 2019 | ZAF | 2009-2017 | Moderate | Moderate | Moderate | Low | Moderate | Low | Low |
| Borgdorff 2018 | KEN | 2010-2016 | Serious | Low | Moderate | Serious | Serious | Moderate | Low |
| Kagaayi 2019 | UGA | 2011-2017 | Moderate | Low | Moderate | Low | Low | Low | Low |
| **F) Repeat cross-sectional cohorts (changes in HIV prevalence ratio)** | | | | | | | | | |
| Auvert 2013 | ZAF | 2007-2011 | Moderate | Low | Low | Low | Low | Low | Low |
| Kagaayi 2019 | UGA | 2011-2017 | Moderate | Low | Moderate | Low | Low | Low | Low |

Legend
- Low
- Moderate
- Serious
- Critical
- No Information

Note:
'S&E Afr' = Seven countries in southern and eastern Africa (BWA, KEN, RWA, TZA, UGA, ZAF, ZMB)

Figure S2: Incidence ratios in community-based studies in Africa without serious risk of bias during circumcision scale-up with studies ordered by A) time period, B) HIV incidence in uncircumcised men, C) average circumcision prevalence during scale-up, and D) average ART prevalence in women during scale-up

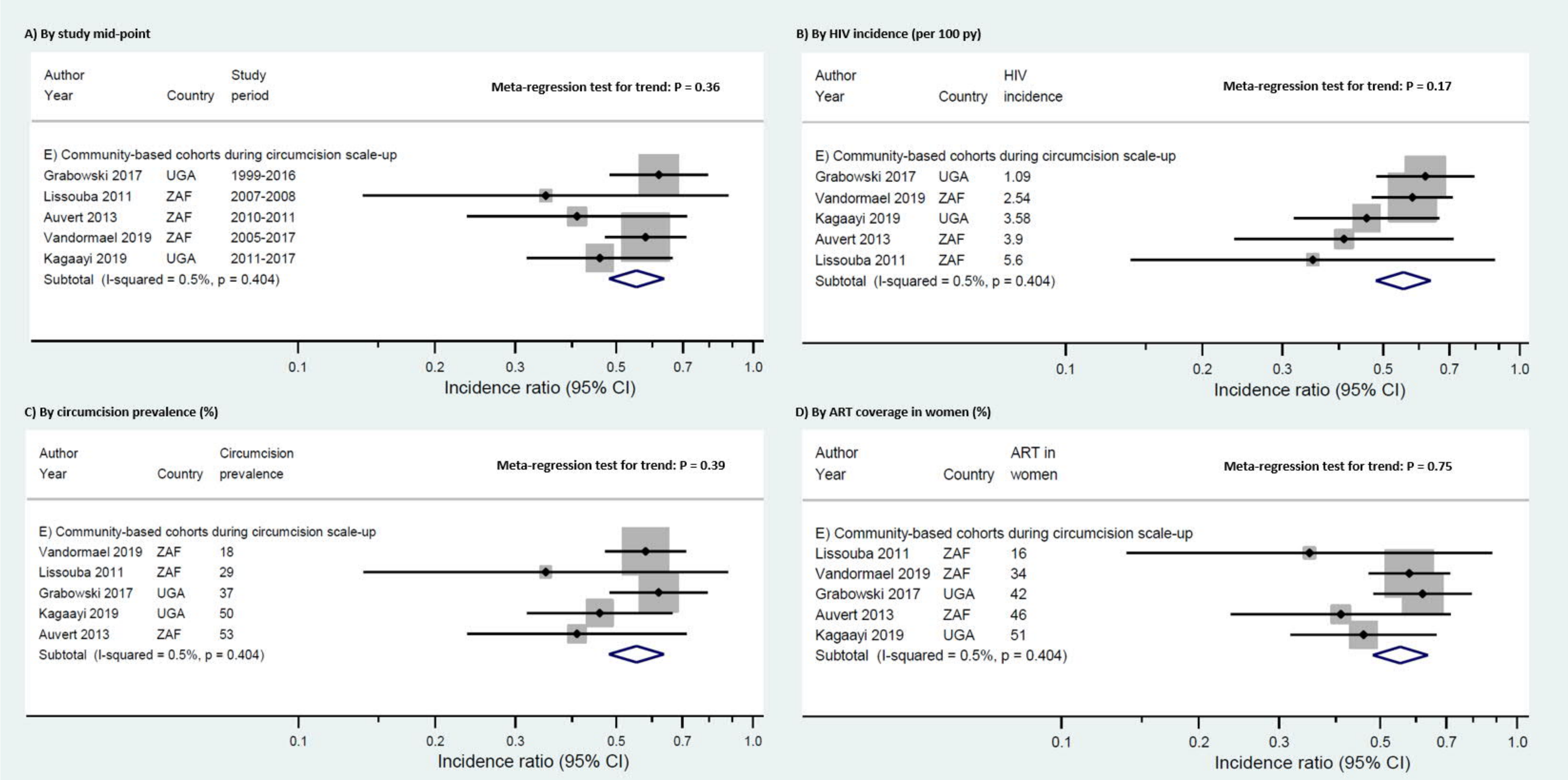

Figure S3: Incidence ratios in observational studies in Africa without serious risk of bias by HIV incidence A) by study type and B) as a single group

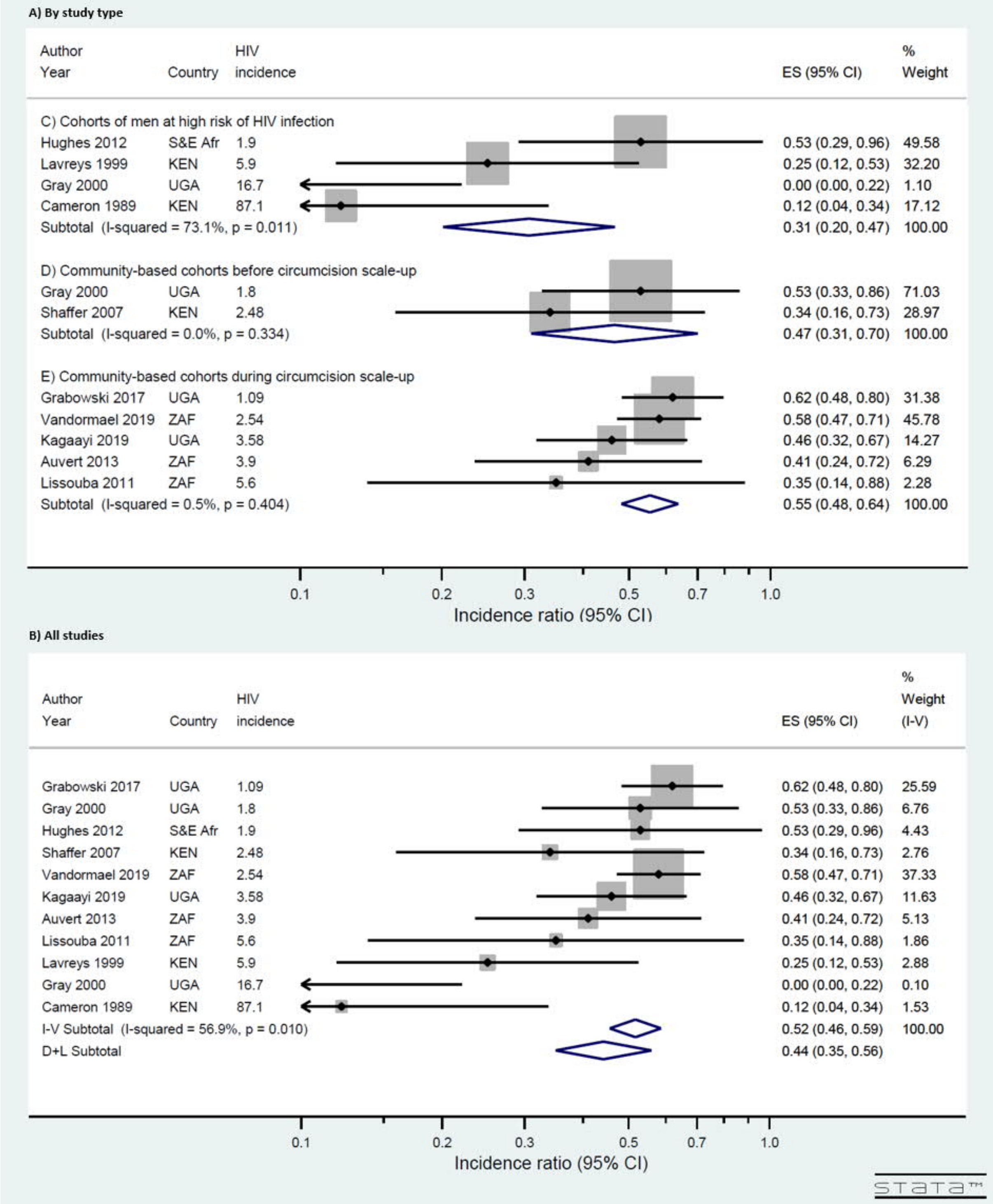

## References

1. Quinn TC, Wawer MJ, Sewankambo N, Serwadda D, Li C, Wabwire-Mangen F, et al. Viral load and heterosexual transmission of human immunodeficiency virus type 1. N Engl J Med. 2000;342:921-9.

2. Gray RH, Kiwanuka N, Quinn TC, Sewankambo NK, Serwadda D, Mangen FW, et al. Male circumcision and HIV acquisition and transmission: cohort studies in Rakai, Uganda. AIDS. 2000;14:2371-81.

3. Baeten JM, Richardson BA, Lavreys L, Rakwar JP, Mandaliya K, Bwayo JJ, et al. Female-to-male infectivity of HIV-1 among circumcised and uncircumcised Kenyan men. J Infect Dis. 2005;191:546-53.

4. Lavreys L, Rakwar JP, Thompson ML, Jackson DJ, Mandaliya K, Chohan BH, et al. Effect of circumcision on incidence of human immunodeficiency virus type 1 and other sexually transmitted diseases: a prospective cohort study of trucking company employees in Kenya. J Infect Dis. 1999;180(2):330-6.

5. Gray RH, Kigozi G, Serwadda D, Makumbi F, Nalugoda F, Watya S, et al. The effects of male circumcision on female partners' genital tract symptoms and vaginal infections in a randomized trial in Rakai, Uganda. AJOG. 2009;200(42):e1-e7.

6. Gray RH, Kigozi G, Serwadda D, Makumbi F, Watya S, Nalugoda F, et al. Male circumcision for HIV prevention in men in Rakai, Uganda: a randomised trial. Lancet. 2007;369(9562):657-66.

7. Mahiane S-G, Legeai C, Taljaard D, Latouche A, Puren A, Peillon A, et al. Transmission probabilities of HIV and herpes simplex virus type 2, effect of male circumcision and interaction: a longitudinal study in a township of South Africa. AIDS. 2009;23(3):377-83.

8. Auvert B, Taljaard D, Lagarde E, Sobngwi-Tambekou J, Sitta R, Puren A. Randomized, controlled intervention trial of male circumcision for reduction of HIV infection risk: the ANRS 1265 Trial. PLoS Med. 2005;2(11):e298.

9. Shaffer DN, Ngetich IK, Bautista CT, Sawe FK, Renzullo PO, Scott PT, et al. HIV-1 incidence rates and risk factors in agricultural workers and dependents in rural Kenya: 36-month follow-up of the Kericho HIV Cohort Study. J AIDS. 2010;53(4):514-21.

10. Shaffer DN, Bautista CT, Sateren WB, Sawe FK, Kiplangat SC, Miruka AO, et al. The protective effect of circumcision on HIV incidence in rural low-risk men circumcised predominantly by traditional circumcisers in Kenya: two-year follow-up of the Kericho HIV Cohort Study. J Acquir Immune Defic Syndr. 2007;45(4):371-9.

11. Mehta SD, Moses S, Parker CB, Agot K, Maclean I, Bailey RC. Circumcision status and incident herpes simplex virus type 2 infection, genital ulcer disease, and HIV infection. AIDS. 2012;26(9):1141-9.

12. Bailey RC, Moses S, Parker CB, Agot K, Maclean I, Krieger JN, et al. Male circumcision for HIV prevention in young men in Kisumu, Kenya: a randomised controlled trial. Lancet. 2007;369(9562):643-56.

13. Santelli JS, Edelstein ZR, Wei Y, Mathur S, Song X, Schuyler A, et al. Trends in HIV acquisition, risk factors and prevention policies among youth in Uganda, 1999-2011. AIDS. 2015;29(2):211-9.

14. Grabowski MK, Serwadda DM, Gray RH, Nakigozi G, Kigozi G, Kagaayi J, et al. HIV prevention efforts and incidence of HIV in Uganda. N Engl J Med. 2017;377(22):2154-66.

15. Kong X, Kigozi G, Ssekasanvu J, Nalugoda F, Nakigozi G, Ndyanabo A, et al. Association of medical male circumcision and antiretroviral therapy scale-up with community HIV incidence in Rakai, Uganda. JAMA. 2016;316(2):182-90.

16. Tomita A, Vandormael AM, Barnighausen T, de Oliveira T, Tanser F. Social disequilibrium and the risk of HIV acquisition: A multilevel study in rural KwaZulu-Natal Province, South Africa. J AIDS. 2017;75(2):164-74.

17. Vandormael A, Akullian A, Siedner M, de Oliveira T, Barnighausen T, Tanser F. Declines in HIV incidence among men and women in a South African population-based cohort. Nat Commun. 2019;10(1):5482.

18. Mehta SD, Moses S, Agot K, Odoyo-June E, Li H, Maclean I, et al. The long-term efficacy of medical male circumcision against HIV acquisition. AIDS. 2013;27(18):2899-907.

19. Gray R, Kigozi G, Kong X, Ssempiija V, Makumbi F, Wattya S, et al. The effectiveness of male circumcision for HIV prevention and effects on risk behaviors in a posttrial follow-up study. AIDS. 2012;26(5):609-15.

20. Cameron DW, Simonsen JN, D'Costa LJ, Ronald AR, Maitha GM, Gakinya MN, et al. Female to male transmission of human immunodeficiency virus type 1: risk factors for seroconversion in men. Lancet. 1989;2(8660):403-7.

21. Reynolds SJ, Shepherd ME, Risbud AR, Gangakhedkar RR, Brookmeyer RS, Divekar AD, et al. Male circumcision and risk of HIV-1 and other sexually transmitted infections in India. Lancet. 2004;363(9414):1039-40.

22. Mehendale SM, Rodrigues JJ, Brookmeyer RS, Gangakhedkar RR, Divekar AD, Gokhale MR, et al. Incidence and predictors of human immunodeficiency virus type 1 seroconversion in patients attending sexually transmitted disease clinics in India. J Infect Dis. 1995;172(6):1486-91.

23. Hughes JP, Baeten JM, Lingappa JR, Magaret AS, Wald A, de Bruyn G, et al. Determinants of per-coital-act HIV-1 infectivity among African HIV-1-serodiscordant couples. J Infect Dis. 2012;205(3):358-65.

24. Kim AA, Parekh BS, Umuro M, Galgalo T, Bunnell R, Makokha E, et al. Identifying risk factors for recent HIV infection in Kenya using a recent infection testing algorithm: Results from a nationally representative population-based survey. PLoS One. 2016;11(5):e0155498.

25. Dandona L, Kumar GA, Lakshmi V, Ahmed GMM, Akbar M, Ramgopal SP, et al. HIV incidence from the first population-based cohort study in India. BMC Infect Dis. 2013;13:327.

26. Lissouba P, Taljaard D, Rech D, Dermaux-Msimang V, Legeai C, Lewis D, et al. Adult male circumcision as an intervention against HIV: an operational study of uptake in a South African community (ANRS 12126). BMC Infect Dis. 2011;11:253.

27. Auvert B, Taljaard D, Rech D, Lissouba P, Singh B, Bouscaillou J, et al. Association of the ANRS-12126 male circumcision project with HIV levels among men in a South African township: evaluation of effectiveness using cross-sectional surveys. PLoS Med. 2013;10(9):e1001509.

28. Borgdorff MW, Kwaro D, Obor D, Otieno G, Kamire V, Odongo F, et al. HIV incidence in western Kenya during scale-up of antiretroviral therapy and voluntary medical male circumcision: a population-based cohort analysis. Lancet HIV. 2018;5(5):e241-e9.

29. Kagaayi J, Chang LW, Ssempijja V, Grabowski MK, Ssekubugu R, Nakigozi G, et al. Impact of combination HIV interventions on HIV incidence in hyperendemic fishing communities in Uganda: a prospective cohort study. Lancet HIV. 2019;6(10):680-7.
